# Detection Efficacy of ^18^F-PSMA-1007 PET/CT in 251 Patients with Biochemical Recurrence of Prostate Cancer After Radical Prostatectomy

**DOI:** 10.2967/jnumed.118.212233

**Published:** 2019-03

**Authors:** Frederik L. Giesel, Karina Knorr, Fabian Spohn, Leon Will, Tobias Maurer, Paul Flechsig, Oliver Neels, Kilian Schiller, Horacio Amaral, Wolfgang A. Weber, Uwe Haberkorn, Markus Schwaiger, Clemens Kratochwil, Peter Choyke, Vasko Kramer, Klaus Kopka, Matthias Eiber

**Affiliations:** 1 Department of Nuclear Medicine, Heidelberg University Hospital, Heidelberg, Germany; 2 German Cancer Consortium (DKTK), Heidelberg, Germany; 3 CCU Nuclear Medicine, German Cancer Research Center (DKFZ), Heidelberg, Germany; 4 Department of Nuclear Medicine, Technical University Munich, Munich, Germany; 5 Department of Urology, Technical University Munich, Munich, Germany; 6 Division of Radiopharmaceutical Chemistry, German Cancer Research Center (DKFZ), Heidelberg, Germany; 7 Department of Radiation Oncology, Technical University Munich, Munich, Germany; 8 Center of Nuclear Medicine, PositronMed, FALP, Santiago, Chile; and; 9 Molecular Imaging Program, National Cancer Institute, Bethesda, Maryland

**Keywords:** ^18^F-PSMA-1007, PET/CT, hybrid imaging, prostate cancer, biochemical recurrence

## Abstract

Prostate-specific membrane antigen (PSMA)–targeted PET imaging recently emerged as a new method for the staging and restaging of prostate cancer. Most published studies investigated the diagnostic potential of ^68^Ga-labeled PSMA agents that are excreted renally. ^18^F-PSMA-1007 is a novel PSMA ligand that has excellent preclinical characteristics and that is only minimally excreted by the urinary tract, a potential advantage for pelvic imaging. The aim of this study was to investigate the diagnostic efficacy of ^18^F-PSMA-1007 for biochemical recurrence (BCR) after radical prostatectomy. **Methods:** From 3 academic centers, 251 patients with BCR after radical prostatectomy were evaluated in a retrospective analysis. Patients who had received second-line androgen deprivation therapy (ADT) or chemotherapy were excluded, but prior first-line ADT exposure was allowed. The median prostate-specific antigen (PSA) level was 1.2 ng/mL (range, 0.2–228 ng/mL). All patients underwent PSMA PET/CT at 92 ± 26 min after injection of 301 ± 46 MBq of ^18^F-PSMA-1007. The rate of detection of presumed recurrence sites was correlated with the PSA level and original primary Gleason score. A comparison to a subset of patients treated previously with ADT was undertaken. **Results:** Of the 251 patients, 204 (81.3%) had evidence of recurrence on ^18^F-PSMA-1007 PET/CT. The detection rates were 94.0% (79/84), 90.9% (50/55), 74.5% (35/47), and 61.5% (40/65) for PSA levels of greater than or equal to 2, 1 to less than 2, 0.5 to less than 1, and 0.2 to less than 0.5 ng/mL, respectively. ^18^F-PSMA-1007 PET/CT revealed local recurrence in 24.7% of patients (*n* = 62). Lymph node metastases were present in the pelvis in 40.6% of patients (*n* = 102), in the retroperitoneum in 19.5% of patients (*n* = 49), and in supradiaphragmatic locations in 12.0% of patients (*n* = 30). Bone and visceral metastases were detected in 40.2% of patients (*n* = 101) and in 3.6% of patients (*n* = 9), respectively. In tumors with higher Gleason scores (≤7 vs. ≥8), detection efficacy trended higher (76.3% vs. 86.7%) but was not statistically significant (*P* = 0.32). However, detection efficacy was higher in patients who had received ADT (91.7% vs. 78.0%) within 6 mo before imaging (*P* = 0.0179). **Conclusion:** ^18^F-PSMA-1007 PET/CT offers high detection rates for BCR after radical prostatectomy that are comparable to or better than those published for ^68^Ga-labeled PSMA ligands.

Biochemical recurrence (BCR) represents a major concern for prostate cancer patients who have undergone primary prostatectomy. The ability to localize sites of recurrent prostate cancer is important for directing salvage therapy with curative intent. At present, only conventional imaging, such as whole-body bone scanning and cross-sectional abdominopelvic contrast-enhanced CT imaging or enhanced MRI, is recommended for detecting recurrence; however, these modalities have very limited sensitivity for recurrent disease (1).

The recent introduction of ^68^Ga-PSMA-11 improved prostate cancer detection in the BCR setting. Some form of the ^68^Ga-labeled prostate-specific membrane antigen (PSMA) ligand tracer has been used for more than 3,000 patients worldwide (2–6). The results have surpassed those obtained using PET/CT with ^18^F-choline, formerly considered the best available PET agent for prostate cancer (7,8). Most impressive has been the ability of ^68^Ga-PSMA PET to detect recurrent disease in patients with very low (0.2–0.5 ng/mL) and low (>0.5–1.0 ng/mL) prostate-specific antigen (PSA) levels. Such detection enables tailored decision making regarding further treatment plans (9).

However, there are several reasons to consider ^18^F-labeled PSMA compounds for BCR. Because of their longer half-life (110 vs. 68 min), radiofluorinated tracers are more practical for centralized production and distribution, leading to cost savings. Furthermore, because ^18^F is cyclotron-produced, it can be produced in larger quantities than ^68^Ga, which is generator-produced in a serial fashion. Another potential advantage of ^18^F is that the lower positron energy of ^18^F than of ^68^Ga improves spatial resolution. Thus, there has been interest in the development of ^18^F-labeled PSMA compounds (10,11).

One new candidate is ^18^F-PSMA-1007, which exhibits rapid blood clearance but only minimal amounts of activity excreted via the urinary tract (11). A high concentration of PET agents in the urinary bladder and ureter can interfere with the diagnosis of recurrent disease around the bladder (11–13) and can particularly hamper the detection of local recurrence in the prostate bed and regional pelvic lymph nodes (13,14).

The purpose of this analysis was to assess the performance characteristics of ^18^F-PSMA-1007 PET/CT for the detection and localization of recurrent disease in a multiinstitutional cohort of patients after radical prostatectomy. Specifically, we aimed to establish the rate of detection of recurrence as a function of the absolute PSA level in BCR patients and to compare this to historical data for ^68^Ga-PSMA-11. Further, we compared the impact of the Gleason score at diagnosis and androgen deprivation therapy (ADT) on the efficacy of ^18^F-PSMA-1007 PET/CT for localizing recurrent prostate cancer.

## MATERIALS AND METHODS

### Study Design and Patient Population

A total of 251 patients, with a median age of 70 y (range, 48–86 y), were included in this retrospective multicenter study (*n* = 139 from Technical University of Munich, Munich, Germany; *n* = 70 from Heidelberg University, Heidelberg, Germany; *n* = 42 from Fundación Arturo López Pérez [FALP], Santiago, Chile). Patient characteristics are summarized in Table 1. A total of 60 patients (23.9%) had received first-line ADT within the last 6 mo before the examination. A total of 110 patients (43.8%) had already undergone salvage radiotherapy (RTx) before ^18^F-PSMA-1007 PET/CT. The mean time between surgery and ^18^F-PSMA-1007 PET/CT was significantly longer in patients without prior salvage RTx than in those with prior salvage RTx (93.4 vs. 57.3 mo) (*P* < 0.0001).

**TABLE 1:** Clinical and Pathologic Characteristics of 251 Patients

Characteristic	No. of patients	Range	Percentage of patients
Median age at PET/CT (y)	70	48–86	
Further treatment			
External radiation after radical prostatectomy	110		43.8
Antihormonal treatment	74		29.5
ADT within 6 mo before imaging	60		23.9
Gleason score			
≤6	13		5.2
7	125		49.8
≥8	85		33.1
Unknown	28		11.2
Pathologic primary tumor staging (pT)			
pT2	74		29.5
pT3	92		36.7
pT4	7		2.8
Unknown	78		31.1
Pathologic regional lymph node staging (pN)			
pN0	123		49.0
pN1	41		16.3
pNx	87		34.7
Positive margin			
R0	80		31.8
R1	53		21.1
Unknown	118		47.0
Median initial PSA level (ng/mL)	10.9	0.6–250	
Salvage radiation therapy before PET/CT	110		43.8
Median time between surgery and PET/CT (mo)	57	1–321	
Median last PSA level before PET/CT (ng/mL)	1.2	0.2–228	

The study was approved by the Ethics Committee of the Technical University Munich, the Ethics Committee of Heidelberg University, and the Regional Ethics Committee of the SSM Oriente, Santiago, Chile. All patients gave written informed consent for anonymized evaluation and publication of their data. All reported investigations were conducted in accordance with the Helsinki Declaration and with local regulations. The serum PSA level at the time of the PET/CT scan was available for all patients. PSA kinetic data could not be reliably obtained for this cohort because of the retrospective nature of the study.

We identified patients who underwent ^18^F-PSMA-1007 PET/CT imaging for recurrent prostate cancer from the databases at the 3 institutions (date range: February 2017–January 2018). Only patients who underwent primary radical prostatectomy with or without salvage radiation and who had PSA levels of greater than or equal to 0.2 ng/mL were selected. Patients who had advanced castration-resistant prostate cancer and who underwent second-line ADT, chemotherapy, or radionuclide therapy (^223^Ra-dichloride PSMA-targeted radioligand therapy) were excluded from the analysis.

### Radiosynthesis, Quality Control, and Application of ^18^F-PSMA-1007

^18^F-PSMA-1007 was synthesized as described previously (15). Reagent kits, the unprotected PSMA-1007 precursor, and the PSMA-1007 reference standard were obtained from ABX. Radiosynthesis was performed as single-step radiofluorination on a modified Nuclear Interface FDG synthesis module (GE TRACERlab FX-FN analog), an ORA NEPTIS plug synthesis module, or an IBA Synthera+ synthesis module using 1.6 mg of PSMA-1007 precursor in dimethyl sulfoxide for 10 min at 80°C. Subsequent purification on 2 stacked solid-phase extraction cartridges (PS-H+ and C18_ec_; Macherey–Nagel), final dilution with phosphate-buffered saline, and sterile filtration yielded ^18^F-PSMA-1007 as a solution ready for injection. High-performance liquid chromatography (HPLC) and thin-layer chromatography (TLC) were performed to test radiochemical and chemical purity, residual solvents were tested using gas chromatography, and the tetrabutylammonium content was determined using a TLC spot test. Further quality control (radionuclidic purity, appearance, pH, endotoxins, sterility, and filter integrity) was done in compliance with current pharmacopoeias. ^18^F-PSMA-1007 was given to patients via an intravenous bolus (mean ± SD, 301 ± 46 MBq; range, 154–453 MBq).

### Imaging Protocol

All patients underwent ^18^F-PSMA-1007 PET/CT at 92 ± 26 min after injection of ^18^F-PSMA-1007.

#### Heidelberg University Hospital

All patients (*n* = 70) were imaged on a Biograph mCT Flow scanner (Siemens Medical Solutions). PET was acquired in the 3-dimensional (3D) mode (matrix, 200 × 200) using FlowMotion (Siemens). The emission data were corrected for randoms, scatter, and decay. Reconstruction was performed with an ordered-subset expectation maximization (OSEM) algorithm (2 iterations and 21 subsets) and a gaussian filter to a transaxial resolution of 5 mm at full width at half maximum (FWHM); attenuation correction was performed using unenhanced low-dose CT data. The CT scans were reconstructed to a slice thickness of 5 mm, an increment of 3–4 mm, and a soft-tissue reconstruction kernel (B30) using CareDose (Siemens).

#### Technical University Munich

All patients (*n* = 139) were examined on a Biograph mCT scanner (Siemens Medical Solutions). A diagnostic CT scan was initially performed in the portal venous phase 80 s after the intravenous injection of an iodinated contrast agent (iomeprol [Imeron 300; Bracco UK Ltd.]) and followed by a PET scan. All patients received diluted oral contrast agent (300 mg of ioxitalamate [Telebrix; Guerbet]) and rectal filling with a negative contrast agent (100–150 mL). All PET scans were acquired in the 3D mode with an acquisition time of 3–4 min/bed position. Emission data were corrected for randoms, dead time, scatter, and attenuation and reconstructed iteratively with an OSEM algorithm (4 iterations and 8 subsets) followed by a postreconstruction smoothing gaussian filter (5 mm at FWHM).

#### FALP, Santiago, Chile

All patients (*n* = 42) were imaged on a Biograph mCT20 scanner (Siemens Medical Solutions). A diagnostic CT scan was performed in the equilibrium phase 60–70 s after the intravenous injection of an iodinated contrast agent (loversol [Optiray 300; Mallinckrodt Pharmaceuticals]), and all patients received water as an oral contrast agent. Subsequent PET scans were acquired in the 3D mode with an acquisition time of 1.5 min/bed position. Emission data were corrected for scatter and attenuation and reconstructed iteratively with an OSEM algorithm (2 iterations and 21 subsets) followed by a postreconstruction smoothing gaussian filter (4 mm at FWHM).

### Image Analysis

All images were interpreted by 2 physicians with double board certifications (radiology and nuclear medicine) in consensus. Focal uptake of ^18^F-PSMA-1007 higher than the surrounding background and not associated with physiologic uptake was considered suggestive of malignancy. Typical pitfalls in PSMA ligand PET imaging (e.g., celiac and other ganglia, fractures, and degenerative changes) were considered (16). All lesions suggestive of recurrent prostate cancer were noted and grouped into local recurrence, lymph node metastases (stratified into pelvic, retroperitoneal, and supradiaphragmatic locations), bone metastases, and other metastases (e.g., lung, liver).

### Statistical Analysis

The detection rate (number of patients with at least 1 positive finding) was plotted against the absolute PSA level. Mann–Whitney *U* tests were used to evaluate differences between single groups (e.g., Gleason score, ADT) and to evaluate differences in PSA levels between groups with pathologic uptake and groups without pathologic uptake. The χ^2^ test was used to compare proportions. All tests were 2-sided, and a level of significance (α) of 5% was used. Statistical analyses were conducted with MedCalc software, version 17.8.6.

## RESULTS

### Radiosynthesis and Quality Control

^18^F-PSMA-1007 was obtained in a radiochemical yield of 50% ± 10% after a total synthesis time of 25–45 min. The radiochemical purity of ^18^F-PSMA-1007 was greater than or equal to 95% (HPLC and TLC), with free ^18^F-fluoride being the major impurity. The content of carrier PSMA-1007 in the final product solution was less than 10 μg/mL. The radiopharmaceutical specifications, in accordance with current pharmacopoeias, were met for all productions of the ^18^F-PSMA-1007 final product solution. No radiolysis of ^18^F-PSMA-1007 was observed up to 8 h after the end of synthesis. No adverse events were associated with ^18^F-PSMA-1007.

### Detection Efficacy

**FIGURE 1.**
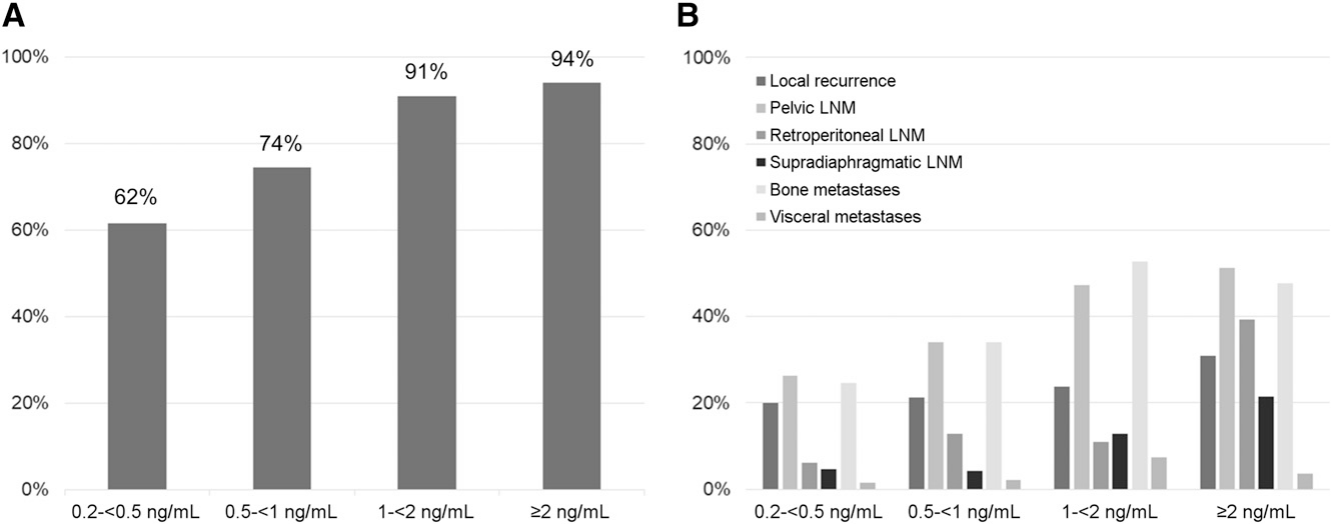
Overall rate of detection for ^18^F-PSMA-1007 PET/CT (A) and relative number of lesions grouped by different regions (B) in relation to PSA levels. LNM = lymph node metastases.

Of the 251 patients, 204 (81.3%) had 1 or more localized areas suggestive of recurrent prostate cancer. The detection efficacies of ^18^F-PSMA-1007 PET/CT were 94.0% (79/84; 95% CI, 0.75–1) for PSA levels of greater than or equal to 2 ng/mL, 90.9% (50/55; 95% CI, 0.67–1) for PSA levels of 1–less than 2 ng/mL, 74.5% (35/47; 95% CI, 0.52–1) for PSA levels of 0.5–less than 1 ng/mL, and 61.5% (40/65; 95% CI, 0.44–0.86) for PSA levels of 0.2–less than 0.5 ng/mL (Fig. 1A). The mean PSA level was significantly lower in patients with negative ^18^F-PSMA-1007 PET/CT findings than in those with positive findings (0.95 ± 1.56 vs. 6.8 ± 22.4 ng/mL) (*P* < 0.0001). There was a trend toward a higher detection rate (86.3% vs. 77.3%) (*P* = 0.07) in patients with prior salvage RTx than in those without prior salvage therapy. However, PSA levels were significantly higher in patients with prior salvage RTx than in those without prior salvage therapy (median, 1.3 vs. 0.98 ng/mL).

### Lesion Location

The different regions involved by recurrent disease are listed in Table 2. Figure 1B shows the percentages of positive lesions for different regions stratified by PSA levels. The relative numbers of lesions indicating local recurrence increased slightly with increasing PSA levels. The numbers increased to 18.5% (12/65), 21.3% (10/47), 23.6% (13/55), and 31.0% (26/84) at PSA levels of 0.2–less than 0.5, 0.5–less than 1, 1–less than 2, and greater than or equal to 2 ng/mL, respectively. Locoregional pelvic lymph node metastases were present in 26.2% (17/65), 34.0% (16/47), 47.2% (26/55), and 51.2% (43/84) of patients at PSA levels of 0.2–less than 0.5, 0.5–less than 1, 1–less than 2, and greater than or equal to 2 ng/mL, respectively.

**TABLE 2:** ^18^F-PSMA-1007 PET/CT Detection of Different Regions Involved by Recurrent Prostate Cancer

Region	No. of patients	Percentage of patients
Local recurrence	62	24.7
Lymph node metastases		
Pelvic	102	40.6
Retroperitoneal	49	19.5
Supradiaphragmatic	30	12.0
Bone metastases	101	40.2
Other (lung, liver) metastases	9	3.6

More than 1 region could be involved per patient.

Distant lymph node metastases were present in only 7.1% (4/56) of patients with very early BCR (PSA levels of 0.2–<0.5 ng/mL). Involvement of supradiaphragmatic nodes was rare in very early BCR and early BCR (PSA levels of 0.2–<0.5 and 0.5–<1 ng/mL, respectively), representing only 4.6% (3/65) and 4.3% (2/47) of cases, respectively. However, involvement of supradiaphragmatic nodes became more common at higher PSA levels (12.7% [7/55] at PSA levels of 1–<2 and 21.4% [18/84] at PSA levels of ≥2 ng/mL). Retroperitoneal lymph node metastases were present in more than 10% of cases at PSA levels of greater than or equal to 0.5 ng/mL (12.8% [6/47], 11.1% [6/54], and 39.3% [33/84] at PSA levels of 0.5–<1, 1–<2, and ≥2 ng/mL, respectively).

**FIGURE 2.**
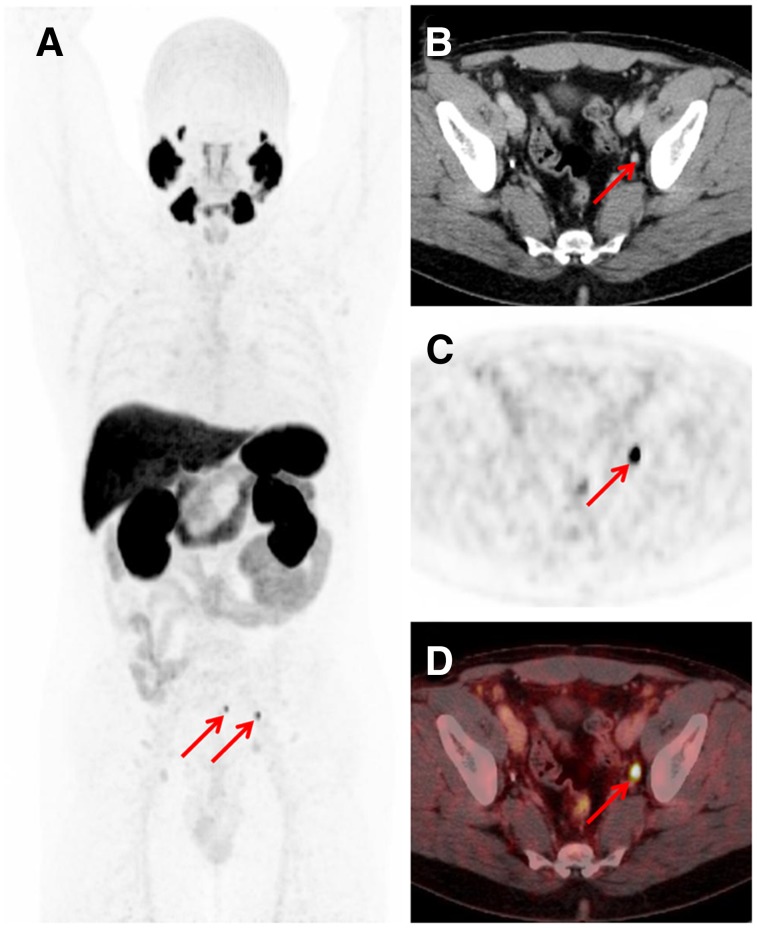
Images from 57-y-old patient after radical prostatectomy (2010; Gleason score of 8; pT3b; pN0), after salvage radiation therapy to prostate bed, and with PSA level rising to 0.43 ng/mL (August 2017). (A) Maximum-intensity projection of ^18^F-PSMA-1007 PET shows 2 intense tracer-associated lesions in left pelvic region (arrows). (B–D) Transaxial PET (C) and fused PET/CT (D) images show high ^18^F-PSMA-1007 uptake in subcentimeter (7-mm) lesions (arrows), as determined by corresponding CT (B). Subsequent radioguided salvage surgery proved malignant nature of lesions.

**FIGURE 3.**
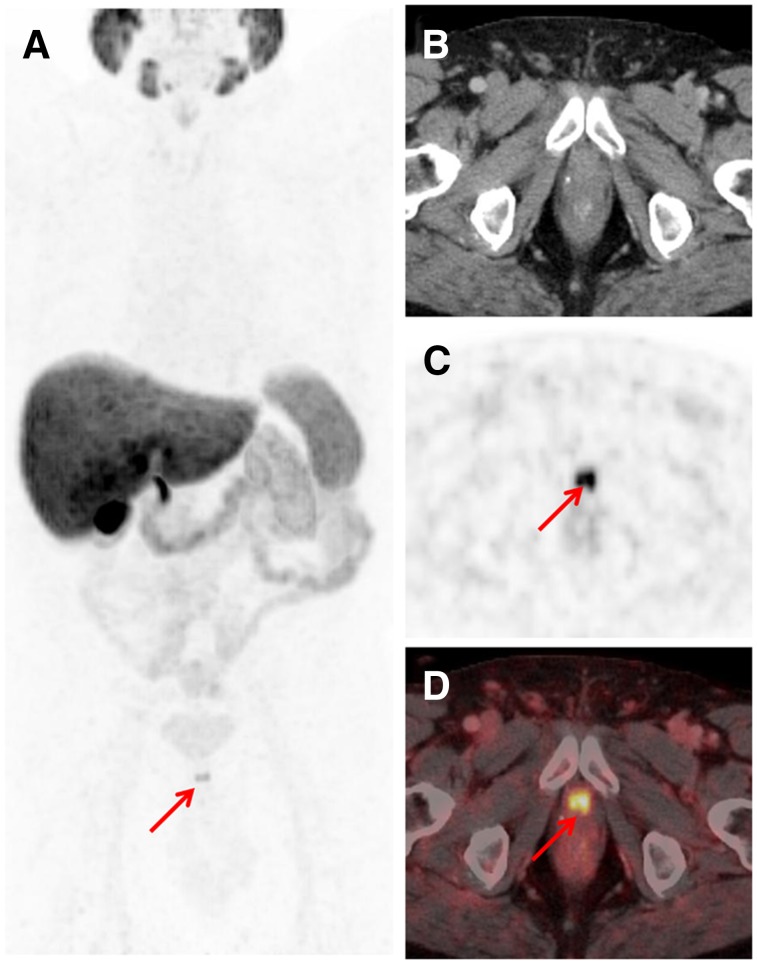
Images from 64-y-old patient after radical prostatectomy (August 2012; Gleason score of 9; pT3b; pN0) and with PSA level rising to 3.9 ng/mL (October 2017). (A) Maximum-intensity projection of ^18^F-PSMA-1007 PET shows intense tracer-associated uptake in lesion (arrow) below bladder. (C and D) It could be localized (arrows) in region of urethral anastomosis using transaxial PET (C) and fused PET/CT (D) images. (B) Corresponding CT image. Postimaging salvage radiation to prostatic fossa was performed in combination with single injection of gonadotropin-releasing hormone analog in October 2017 and resulted in drop in PSA level to below detection threshold (<0.07 ng/mL; last measurement in February 2018).

**FIGURE 4.**
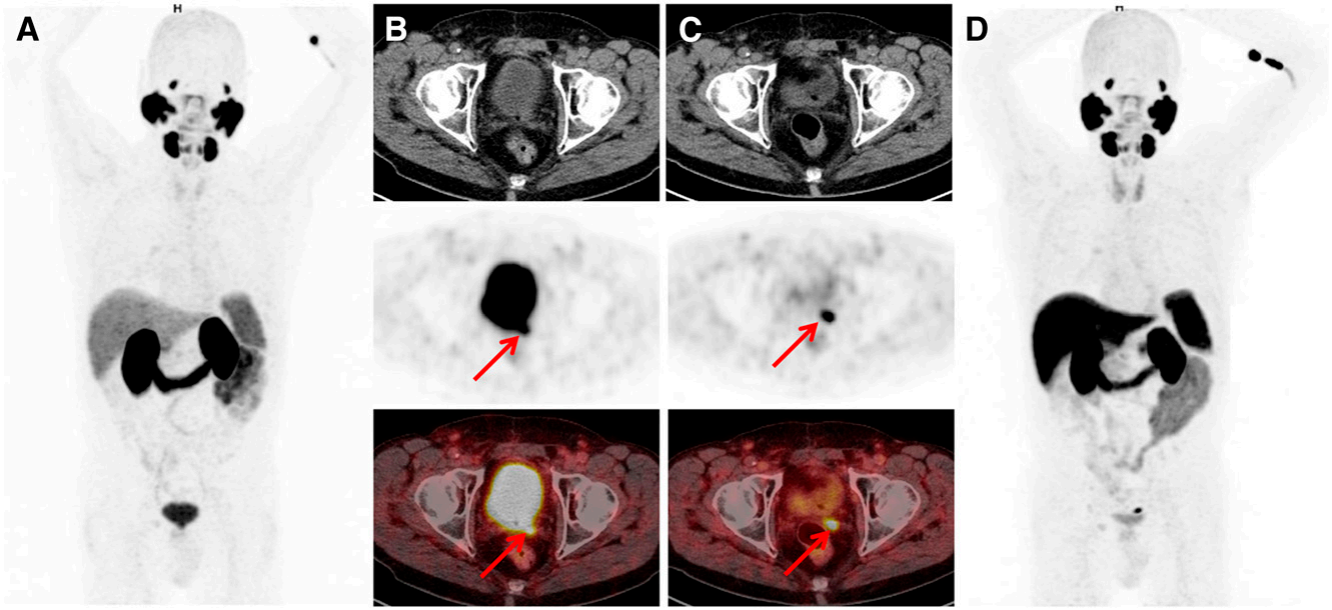
Images from 76-y-old patient after radical prostatectomy (2006; Gleason score of 7b; pT3a; pN0) and with PSA level slowly rising to 0.78 ng/mL (October 2017). (A and B) Patient underwent primarily ^68^Ga-PSMA-11 PET/CT, which resulted in suggestion of local recurrence (arrows). No definite diagnosis could be made on basis of adjacent high activity retention in urinary bladder. (C and D) Subsequent ^18^F-PSMA-1007 PET/CT 3 mo later clearly depicted PSMA ligand uptake in left seminal vesicle (arrows) with high contrast and very low retention in bladder.

Interestingly, findings indicating bone metastases were already present in a considerable number of patients at low PSA levels. Rates of bone metastases increased with increasing PSA levels: 24.6% (16/65), 34.0% (16/47), 52.7% (29/55), and 47.6% (40/84) at PSA levels of 0.2–less than 0.5, 0.5–less than 1, 1–less than 2, and greater than or equal to 2 ng/mL, respectively. Visceral lesions were uncommon in all patient groups: 1.5% (1/65), 2.1% (1/47), 7.3% (4/55), and 3.6% (3/84) at PSA levels of 0.2–less than 0.5, 0.5–less than 1, 1–less than 2, and greater than or equal to 2 ng/mL, respectively. Figures 2–4 showcase examples of different lesion types detected by ^18^F-PSMA1007 PET/CT.

### Influence of Antiandrogen Therapy and Primary Histologic Differentiation

In our cohort, PSMA PET efficacy was statistically higher in patients with prior ADT (*P* = 0.0179). Lesions were detected in 91.7% (55/60) of patients with prior exposure to ADT but in only 78.0% (149/191) of patients with no therapy. PSA levels trended higher in patients with prior ADT (mean ± SD, 8.0 ± 13.9 vs. 5.0 ± 22.0 ng/mL; median, 2.3 vs. 1.0), but this finding was not statistically significant (*P* = 0.32). A similar trend (*P* = 0.08) toward higher detection rates in patients with ADT than in those without ADT was found when the analysis was restricted to patients with PSA levels of less than 2 ng/mL. The corresponding detection rates were 88.5% (23/26) in patients with ADT and 72.3% (102/141) in patients without ADT, respectively.

With respect to the positivity of a PSMA scan versus the original Gleason score of the primary tumor, ^18^F-PSMA-1007 PET/CT findings were positive in 76.3% (106/139) of patients with Gleason scores of less than or equal to 7 and in 86.7% (72/83) of patients with Gleason scores of greater than or equal to 8 (*P* = 0.06). Interestingly, there was no difference in mean PSA levels between these 2 groups (mean PSA level, 4.1 ± 14.8 vs. 8.1 ± 28.7 ng/mL) (*P* = 0.17).

## DISCUSSION

Cancer relapse after radical prostatectomy is common and is denoted by biochemical failure, defined as a rise in the PSA level to 0.2 ng/mL or higher (17). Localizing the recurrence can affect treatment decisions, as local recurrence can be treated with focal radiation, whereas distant metastases require more systemic therapies. Conventional imaging modalities (traditional bone scan, CT, MRI, and other forms of PET) are notoriously insensitive for early recurrent disease (4–6) and especially for the detection of lymph node and distant metastases, which have the greatest impact on patient management (18). Therefore, the goal of the present study was to evaluate ^18^F-PSMA-1007 PET/CT for identifying sites of recurrence.

Several other PET agents have been introduced for detecting sites of BCR. Detection rates vary widely; between 34% and 88% for ^11^C-choline, 43%–79% for ^18^F-fluoromethylcholine, and 59%–80% for ^11^C-acetate (7,19–24). With the introduction of ^68^Ga-PSMA ligand PET/CT, much improved rates of detection, especially in patients with very low and low PSA levels, have been observed. Recently, another ^18^F-labeled PSMA radioligand, 2-(3-{1-carboxy-5-[(6-[^18^F]fluoropyridine-3-carbonyl)-amino]-pentyl}-ureido)-pentanedioic acid (^18^F-DCFPyL), was introduced and is undergoing prospective evaluation. All of these agents are primarily excreted via the urinary route (25).

We recently evaluated ^18^F-PSMA-1007 in a small number of patients with BCR (26). It showed favorable detection rates in PSMA-positive prostate cancer patients but was only minimally excreted by the urinary tract. Of importance is the fact that ^18^F-PSMA-1007 can be produced in high radiochemical yields (50% ± 10%) on a variety of automated radiosynthesizers by direct radiofluorination of the unprotected radiolabeling precursor and composition of the final injection solution by simple solid-phase extraction without (semi)preparative radio-HPLC separation (15). This simple radiosynthesis can be easily set up as good manufacturing practices–compliant production and results in activity amounts per batch that not only fulfill on-site clinical needs but also can be used sustainably for distributing ^18^F-PSMA-1007 to satellite PET/CT centers.

**FIGURE 5.**
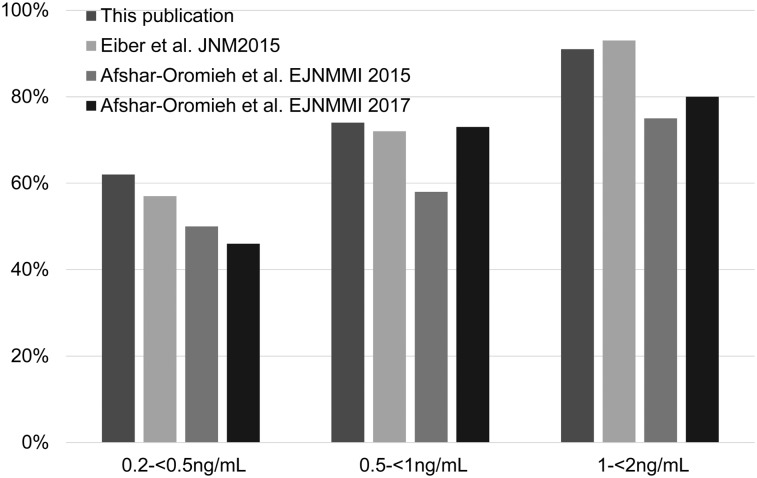
Comparison of rates of detection for ^18^F-PSMA-1007 and ^68^Ga-PSMA-11 derived from different studies.

The straightforward good manufacturing practices–compliant radiosynthesis of ^18^F-PSMA-1007 without HPLC separation (feasible on different automated radiosynthesizers) bodes well for the economic production of the agent. Indeed, the ease with which it was synthesized contributed to the ability to select 251 patients with a highly specific indication from an even larger patient cohort relatively quickly at 3 institutions. Although the present study was retrospective in nature, it nevertheless provides a good basis for future prospective trials, as it indicates that the performance of ^18^F-PSMA-1007 is equal to or better than that of other PSMA PET agents. For instance, the results suggest that ^18^F-PSMA-1007 PET/CT exhibits a higher detection rate in patients with very low PSA levels (0.2–0.5 ng/mL) compared with literature reports for ^68^Ga-PSMA-11 (62% vs. 46%–58%) (Fig. 5) (2,3). Of note, early treatment of these patients has been shown to result in better outcomes (27,28); therefore, more sensitive detection is an advantage. At higher PSA levels, detection rates for ^18^F-PSMA-1007 and ^68^Ga-PSMA-11 appear to be similar—for example, PSA levels of 0.5–1 ng/mL (74% for ^18^F-PSMA-1007 PET/CT vs. 73% for ^68^Ga-PSMA-11 (2,3)). A bar graph comparing published detection rates for ^68^Ga-PSMA-11 and detection rates for ^18^F-PSMA-1007 from the present study is shown as Figure 5.

The slightly improved rate of detection of ^18^F-PSMA-1007 at low PSA levels might also be related to the different energy profiles of the positron emitters ^18^F and ^68^Ga; the theoretically achievable resolution of ^18^F is higher than that of ^68^Ga, particularly in human PET systems (24). Therefore, it could be assumed that ^18^F-labeled PSMA ligands might improve the sensitivity of detection of very small tumors. A more likely source of the advantage for ^18^F-PSMA-1007 is that it is cleared via hepatobiliary excretion. This route of clearance might be diagnostically advantageous, in particular, for detecting local recurrence and locoregional pelvic lymph node metastases (29–31). We do not believe that strong conclusions should be drawn from the higher rate of detection in patients with prior ADT exposure. It is likely that these patients had more advanced disease than the untreated patients, as they showed rising PSA levels despite ADT and possibly radiation therapy. In addition, the data presented indicate higher PSA levels in the group of patients with ADT than in the group of patients without ADT within 6 mo before imaging—a finding that could constitute a confounding factor. The role of ADT in PSMA ligand PET uptake is highly controversial. At the cellular level, ADT initially increases PSMA expression. However, it is unclear what happens in patients receiving ongoing treatment, as this leads to a decrease in the number of tumor cells and the relative magnitudes of these individual effects likely vary according to whether the tumor is castrate sensitive or castrate resistant.

A limitation of the present study and most PSMA PET studies is the lack of histopathologic confirmation of the detected lesions. Many detected recurrences and nodes are quite small and difficult to biopsy. However, when histopathologic validation is available, it shows a very high positive predictive value of PSMA ligand PET agents. Results from a recent ^18^F-PSMA-1007 study that included histopathologic confirmation showed a high correlation between PSMA-positive lesions and PSMA-positive histopathologic findings for primary tumors and locoregional metastases (sensitivity, 94.7%) (11). Another limitation of the present study is that it was retrospective in nature and therefore lacked some potentially interesting information about the effects of PSA kinetics and patient outcomes. We hope that prospective studies will be performed in the near future to overcome these limitations,. Notably, in the present study, PSA levels of equal to or greater than 0.2 ng/mL were defined as being indicative of BCR. This cutoff is the most accepted one despite extensive discussions in the literature (32).

## CONCLUSION

^18^F-PSMA-1007 PET/CT demonstrated a high detection rate for patients with BCR after radical prostatectomy. ^18^F-PSMA-1007 PET/CT could improve patient management by correctly identifying sites of recurrence early in the course of the disease. Perhaps because of its alternate route of excretion—which bypasses the urinary tract—^18^F-PSMA-1007 shows specific advantages for detecting local recurrence and locoregional nodes, which are generally more prevalent at very low PSA levels.

## DISCLOSURE

This work was partly funded by a grant of the Federal Ministry of Education and Research (BMBF), project ProstaPET (2U2WTZKOREA-021; no. 01DR17031A). Matthias Eiber was supported by SFB 824 (DFG Sonderforschungsbereich 824, Project B11) from the Deutsche Forschungsgemeinschaft, Bonn, Germany. A patent application for PSMA-617 was made by Klaus Kopka and Uwe Haberkorn. A patent application for PSMA-1007 was made by Uwe Haberkorn, Frederik L. Giesel, and Klaus Kopka. Frederik L. Giesel is a scientific consultant/advisor to ABX. No other potential conflict of interest relevant to this article was reported.
